# Quotient of Waist Circumference and Body Mass Index: A Valuable Indicator for the High-Risk Phenotype of Obesity

**DOI:** 10.3389/fendo.2021.697437

**Published:** 2021-05-31

**Authors:** Xiao-cong Liu, Yu Huang, Kenneth Lo, Yu-qing Huang, Ji-yan Chen, Ying-qing Feng

**Affiliations:** Department of Cardiology, Guangdong Cardiovascular Institute, Guangdong Provincial People’s Hospital, Guangdong Academy of Medical Sciences, Guangzhou, China

**Keywords:** body mass index, waist circumference, waist-BMI ratio, obesity, mortality

## Abstract

**Objective:**

Measuring the body mass index (BMI) or waist circumference (WC) alone is insufficient for assessing possible health risks due to obesity. This study aimed to investigate whether the quotient of WC and BMI can be used as a proxy of the high-risk phenotype of obesity.

**Methods:**

Data for analysis were derived from the National Health and Nutrition Examination Survey (NHANES 1999-2014). The Waist-BMI Ratio was defined as WC divided by BMI. The associations between Waist-BMI Ratio and mortality were estimated using Cox regression models. Restricted cubic spline and two-piecewise linear regression models were used to identify non-linear relationships. The discriminative abilities of different anthropometric measures were compared using receiver operating characteristic curves (ROC).

**Results:**

This study is based on data from 35557 adults (51.1% female, mean age 44.9 years). During an average follow-up of 101.8 months, 3680 participants died, including 807 of cardiovascular causes. In fully adjusted models, Waist-BMI Ratio was independently associated with overall (hazard ratio [HR], 1.78; 95% confidence interval [CI], 1.48-2.13) and cardiovascular (HR, 1.77; 95% CI, 1.25-2.52) mortality. Spline analyses revealed that dose-response relationships existed between Waist-BMI Ratio and death. The mortality risk rises dramatically above the cut-off point of the Waist-BMI Ratio (HR, 3.22; 95% CI, 2.43-4.26 for overall mortality and HR, 3.07; 95% CI, 1.71-5.52 for cardiovascular mortality). ROC curve analysis suggested that Waist-BMI Ratio was a better discriminator of mortality (AUC 0.637 for overall and 0.639 for cardiovascular mortality) than BMI, WC, and waist-to-height ratio (Delong’s test all P <0.001).

**Conclusions:**

Waist-BMI Ratio was independently associated with overall and cardiovascular mortality in a J-shaped pattern, offering an immense potential risk marker for obesity in the clinical setting.

## Introduction

Obesity has been recognized as one of the three gravest threats to human health and survival (1). It is responsible for 40% of cases of cardiovascular disease, most cases of type 2 diabetes, and more than 10% of gastrointestinal as well as urogenital cancer (2). Although increasing attention is being given to the problem, the prevalence of overweight and obesity has doubled since 1980 around the world and has shown a continuous increase in most countries (3). To reverse this growth and reduce the healthcare burden, accurate assessments of obesity are essential in order to identify high-risk individuals and thus implement appropriate behavior modifications and early therapeutic intervention.

Although multiple methods have been developed to assess obesity, each method has its own strengths and weaknesses. Imaging-based methods, such as dual x-ray absorptiometry and magnetic resonance imaging, can offer precise assessments and body fat quantifications (4). However, these technologically complex methods are too expensive and time-consuming for regular screening. The body mass index (BMI) is a simple anthropometric measure that has been routinely used to identify overweight individuals and estimate body fat (5). Nevertheless, BMI fails to describe body fat distribution and distinguish lean mass from fat mass, which has sparked the controversy related to “obesity paradox” (6–8). Prior studies have demonstrated that visceral adipose tissue (VAT) has an adverse impact on the cardiovascular and metabolic systems (4, 9), while certain types of peripheral fat could actually be metabolically, immunologically, and mechanically protective, and act as a cushion for potential health shocks (10, 11). Waist circumference (WC) and waist-to-height ratio (WtHR) are more accurate anthropometric measures of VAT but there are limitations with the use of either measure alone (12, 13).

Integration of BMI with WC/WtHR in clinical assessment has been recommended, as it may be able to discriminate the higher-risk phenotype of obesity (14, 15); it has been generally implemented by BMI stratification or introducing both variables into regression models. Studies have revealed that WC is positively correlated with mortality after adjustment for BMI, and patients with higher WtHR and lower BMI are at the highest risk of developing cardiovascular events (16–18). However, no study has yet focused on whether the quotient of WC and BMI (Waist-BMI Ratio) can be used to distinguish the high-risk phenotype of obesity. In this study, we evaluated the relationship of the Waist-BMI Ratio with cause-specific mortality and compared its predictive capacity with traditional anthropometric measures, including BMI, WC, and WtHR.

## Materials and Methods

### Study Design and Participants

**Figure 1:**
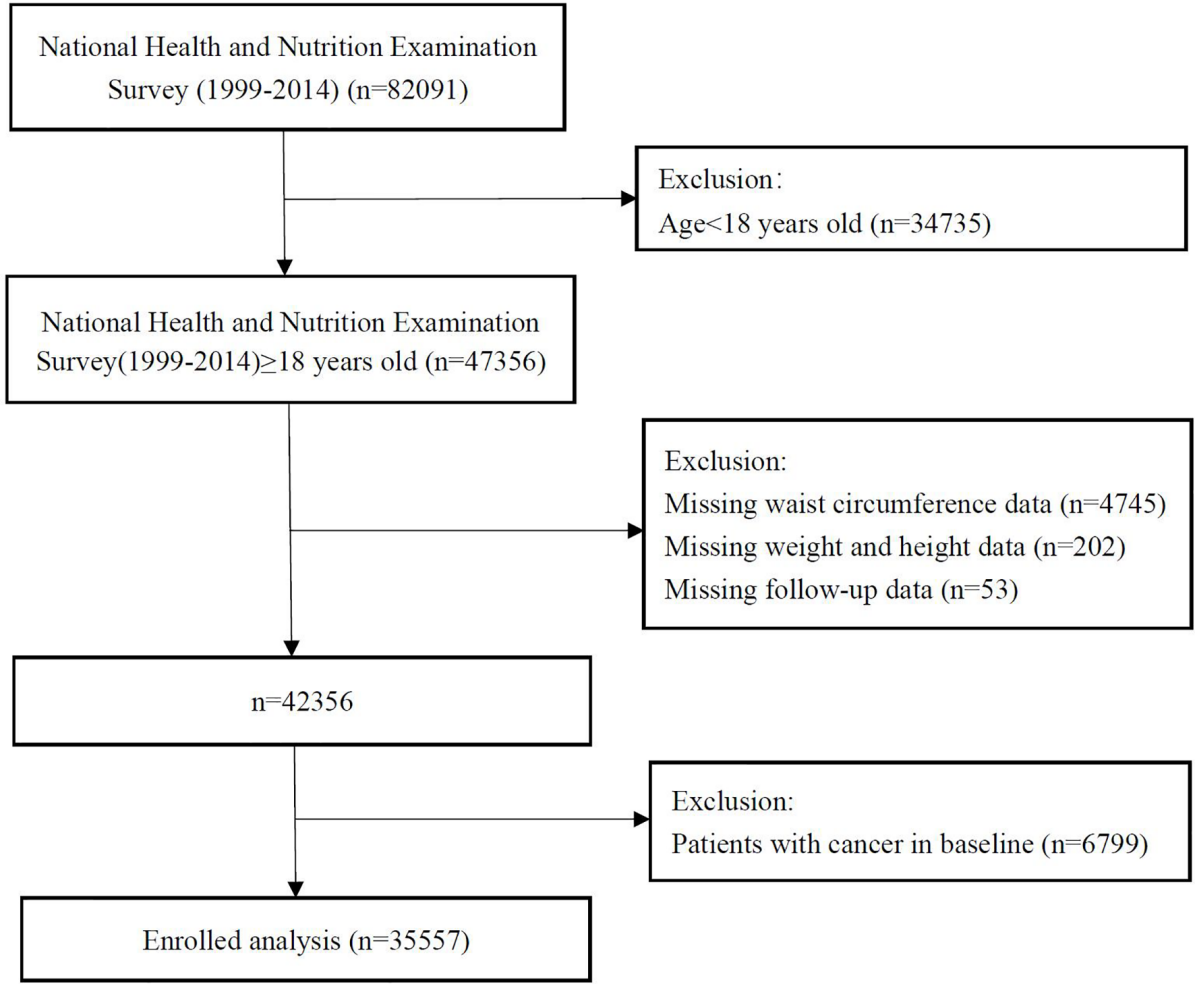
Study cohort.

The study population was drawn from the National Health and Nutrition Examination Survey (NHANES). The NHANES is a national, cross-sectional, multistage, probability sampling survey used to provide representative samples of the non-institutionalized US resident population (19). The survey protocols were approved by the ethics review board of the National Center for Health Statistics, and informed consent was obtained from all participants. Our analysis involved all participants who were ≥ 18 years old from NHANES 1999-2000 to NHANES 2013-2014. After excluding those with missing data and with cancer at baseline, the final study size comprised 35557 individuals (**Figure 1**).

### Anthropometric Measurements

Height, weight, and waist circumference were measured by trained personnel following a standard protocol (19). Waist circumferences were measured at the uppermost lateral border of the right ilium to the nearest 1 millimeter. BMI was calculated as weight in kilograms divided by height in meters squared. Waist-to-height ratio was calculated as waist circumference in centimeters divided by height in centimeters. Waist-BMI Ratio was defined as waist circumference in centimeters divided by BMI:

$$Waist-BMI\;ratio=waist\;circumference\;(cm)/body\;mass\;index\;(kg/m^{2}).$$

### Outcomes

Mortality status for NHANES participants was ascertained through probabilistic record matching with the National Death Index (20). The primary outcome of this study was overall mortality and the secondary outcome was cardiovascular mortality, assessed *via* underlying causes of death with International Classification of Diseases, 10th Revision (ICD-10) codes I00–I09, I11, I13, I20–I51, and I60–I69 (21).

### Covariates

Demographic information including age, gender, and race/ethnicity (categorized as non-Hispanic white, non-Hispanic black, other Hispanic, Mexican American, and other) was collected by standard questionnaires. Past medical history, prescription medication use, and smoking status were self-reported. Blood pressure was measured by trained personnel. Lipid profile data were derived from laboratory measurements. The estimated glomerular filtration rate (eGFR) was calculated using the Modification of Diet in Renal Disease formula (22). Cardiovascular disease (CVD) was defined as self-reported coronary artery disease, angina, heart attack, or stroke. Diabetes was defined as a self-reported history of diabetes, diabetes medication use, fasting blood glucose level of at least 7.0 mmol/L, or a hemoglobin A1c (HbA1c) level of at least 6.5% (23). Hypertension was defined as a self-reported history of hypertension, receiving blood pressure control medication, systolic blood pressure of at least 140 mmHg, or diastolic blood pressure of at least 90 mmHg (24).

### Statistical Analysis

To account for the complex survey design of NHANES, appropriate sampling weights were used to reconstitute data on the US non-institutionalized population. Participants were divided into quintile groups by the Waist-BMI Ratio. The mean or percentage, with standard error (SE), was provided by quintile groups. The linear trend for baseline characteristics was tested by linear or logistic regression whenever appropriate. Kaplan-Meier survival analyses were performed to evaluate the incidence rate of mortality among Waist-BMI Ratio groups, and discrepancies among groups were evaluated by log-rank test. Three sets of Cox proportional hazard models were constructed to evaluate associations with mortality from the date of medical examination to the date of either death or censoring (December 31, 2015), whichever came first. Tests for linear trends were also performed, by entering the mean value of each quintile group of Waist-BMI Ratio as a continuous variable. Restricted cubic regression splines were employed to examine the associations of different anthropometric measures with mortality. We used a two-piecewise linear regression model to evaluate the nonlinear relationships between Waist-BMI Ratio and mortality, and the optimal cut-off points were set by testing all possible values and selecting the cut-off values with the highest likelihood. The difference between one-line linear regression models and two-piecewise linear regression models was assessed by means of logarithmic likelihood ratio tests. To compare the effects of different anthropometric measures, we used the receiver operating characteristic (ROC) curve and the area under the ROC curve (AUC) to identify the ability of baseline BMI, WC, WtHR, and Waist-BMI Ratio to predict mortality events. The statistical significance of the differences in AUC was calculated with Delong’s test. All analyses were conducted in R version 4.0.3 (R Foundation for Statistical Computing), including the rms, survey, pROC, and survminer packages. Two-sided *P* < 0.05 was regarded as a significant difference.

## Results

### Baseline Characteristics

Data were analyzed for 35557 adults from the continuous NHANES survey (1999–2014); 51.1% were females, and the mean age was 44.9 years. Baseline characteristics stratified by Waist-BMI Ratio are presented in **Table 1**. Briefly, during the average follow-up period of 101.8 months, 3680 deaths were recorded and 807 of them were attributed to cardiovascular disease. The ranges of Waist-BMI Ratio were: Q1: 1.80-3.20; Q2: 3.20-3.39; Q3: 3.39-3.56; Q4:3.56-3.75; Q5: 3.75-5.56. Individuals in the upper Waist-BMI Ratio quintiles tended to be older, male, and have higher rates of smoking and CVD. High density lipoprotein (HDL) cholesterol levels and mortality risk tended to be higher with the increase of Waist-BMI Ratio while eGFR and WtHR were decreased with increasing Waist-BMI Ratio quintiles (all *P* for trend < 0.001).

**Table 1:** Baseline characteristics according to Waist-BMI Ratio quintiles.

Variables	Total	Waist-BMI Ratio					*P* for trend
		Q1	Q2	Q3	Q4	Q5	
Number	35557	7112	7111	7111	7112	7111	
Age, years	44.9 (0.19)	42.7 (0.26)	42.4 (0.27)	44.1 (0.25)	45.7 (0.31)	49.4 (0.31)	<0.001
Gender-female, %	51.1 (0.27)	78.8 (0.60)	56.5 (0.86)	44.9 (0.67)	40.2 (0.62)	37.2 (0.85)	<0.001
Race, %							
Mexican American	8.6 (0.61)	10.5 (0.80)	11.1 (0.86)	9.4 (0.69)	7.6 (0.54)	4.7 (0.43)	<0.001
Other Hispanic	5.7 (0.54)	7.7 (0.77)	6.8 (0.72)	5.5 (0.54)	5.1 (0.54)	3.5 (0.46)	<0.001
Non-Hispanic White	67.7 (1.17)	56.6 (1.52)	62.5 (1.51)	68.2 (1.26)	72.1 (1.08)	78.2 (1.04)	<0.001
Non-Hispanic Black	11.6 (0.64)	20.6 (1.14)	13.5 (0.79)	10.2 (0.65)	7.8 (0.48)	6.6 (0.44)	<0.001
Other	6.4 (0.32)	4.6 (0.39)	6.1 (0.47)	6.7 (0.49)	7.5 (0.46)	7.0 (0.49)	<0.001
Smoking, %	46.2 (0.56)	36.7 (0.70)	42.8 (0.85)	45.6 (0.80)	49.9 (0.97)	55.4 (1.04)	<0.001
Systolic blood pressure, mmHg	121.6 (0.18)	122.5 (0.28)	121.1 (0.29)	121.1 (0.24)	121.3 (0.28)	122.2 (0.33)	0.548
Diastolic blood pressure, mmHg	71.0 (0.16)	71.7 (0.24)	71.2 (0.25)	71.1 (0.23)	70.8 (0.22)	70.2 (0.21)	<0.001
eGFR, mg/min/1.73m^2^	87.6 (0.32)	90.5 (0.49)	89.1 (0.43)	86.7 (0.45)	86.2 (0.45)	85.9 (0.48)	<0.001
Total cholesterol, mg/dL	197.9 (0.37)	197.0 (0.59)	198.2 (0.66)	198.7 (0.69)	199.0 (0.65)	196.5 (0.74)	0.903
HDL-cholesterol, mg/dL	52.7 (0.16)	51.2 (0.27)	51.2 (0.28)	51.9 (0.25)	53.6 (0.26)	55.4 (0.26)	<0.001
Body Measures							
Waist circumference, cm	97.4 (0.18)	107.2 (0.36)	98.9 (0.30)	96.4 (0.24)	93.9 (0.23)	91.3 (0.20)	<0.001
Body mass index, kg/m2	28.4 (0.07)	36.1 (0.14)	30.0 (0.09)	27.7 (0.07)	25.8 (0.06)	23.3 (0.05)	<0.001
Waist-Height Ratio	0.58 (0.001)	0.66 (0.002)	0.59 (0.002)	0.57 (0.001)	0.55 (0.001)	0.53 (0.001)	<0.001
Waist-BMI Ratio	3.48 (0.003)	2.99 (0.003)	3.30 (0.001)	3.48 (0.001)	3.65 (0.001)	3.93 (0.003)	<0.001
Comorbidities, %							
Diabetes	11.2 (0.22)	14.9 (0.54)	11.6 (0.54)	10.9 (0.45)	8.9 (0.39)	10.2 (0.43)	<0.001
Hypertension	36.1 (0.46)	41.7 (0.86)	34.7 (0.77)	33.7 (0.70)	34.0 (0.70)	36.8 (0.84)	<0.001
Cardiovascular disease	6.4 (0.19)	5.1 (0.31)	5.2 (0.36)	6.1 (0.33)	6.9 (0.39)	8.9 (0.43)	<0.001
Medicine use, %							
Antihypertensive drugs	20.7 (0.4)	25.7 (0.81)	18.9 (0.64)	19.1 (0.60)	18.5 (0.64)	21.7 (0.70)	<0.001
Hypoglycemic agents	6.0 (0.17)	8.2 (0.41)	6.3 (0.42)	5.5 (0.31)	4.5 (0.29)	5.5 (0.27)	<0.001
Lipid-lowering drugs	11.1 (0.28)	9.3 (0.49)	9.7 (0.47)	11.1 (0.50)	12.0 (0.51)	13.4 (0.58)	<0.001
Antiplatelet drugs	1.2 (0.08)	1.0 (0.17)	0.9 (0.11)	1.0 (0.17)	1.3 (0.16)	2.0 (0.19)	<0.001
Outcomes, %							
Cardiovascular disease mortality	1.5 (0.07)	0.9 (0.11)	1.0 (0.13)	1.1 (0.11)	1.6 (0.17)	2.6 (0.22)	<0.001
Overall mortality	7.2 (0.21)	5.0 (0.33)	4.8 (0.28)	6.0 (0.31)	7.7 (0.39)	12.3 (0.52)	<0.001

Q, quintiles; eGFR, estimated glomerular filtration rate; HDL, high density lipoprotein.

Values are mean or percent with standard error.

P for trend was tested by linear or logistic regression.

### The Association of Waist-BMI Ratio and Other Anthropometric Measures With Overall and Cardiovascular Mortality

**Figure 2:**
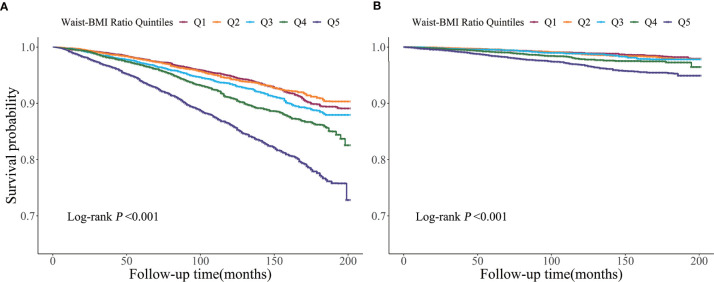
Kaplan-Meier survival curve for overall **(A)** and cardiovascular **(B)** mortality by Waist-BMI Ratio quintiles.

As shown in **Table 2**, the crude incidence rate per 1000 person-years of overall and cardiovascular mortality rose dramatically with increasing Waist-BMI Ratio categories. Kaplan-Meier curves for mortality showed significant differences among the Waist-BMI Ratio quintile groups (**Figure 2**, both log-rank *P* < 0.001). After adjustment for all covariables, including age, gender, race, smoking, systolic blood pressure, HDL-cholesterol, total cholesterol, eGFR, hypertension, diabetes, CVD, antihypertensive drugs, hypoglycemic agents, lipid-lowering drugs, and antiplatelet drugs, Waist-BMI Ratio was positively associated with overall (hazard ratio [HR], 1.78; 95% confidence interval [CI], 1.48-2.13; *P* < 0.001) and cardiovascular (HR, 1.77; 95% CI, 1.25-2.52; *P* = 0.001) mortality as a continuous linear variable. When using the lowest Waist-BMI Ratio quintile (Q1) as the reference, significant association with overall death can be seen for the upper quintile (HR, 1.40; 95% CI, 1.17-1.67; *P* < 0.001). However, the relationship between the highest Waist-BMI Ratio quintile (Q5) and cardiovascular death was not significant (HR, 1.34; 95% CI, 0.95-1.88; *P* = 0.094).

**Table 2:** Multivariate Cox regression analysis of Waist-BMI Ratio with cause-specific mortality.

	Overall mortality				Cardiovascular mortality			
	Event rate/1000 person-years	Model I	Model II	Model III	Event rate/1000 person-years	Model I	Model II	Model III
Waist-BMI Ratio								
As continuous variables	13.65	3.55 (3.08, 4.09) <0.001	1.60 (1.34, 1.90) <0.001	1.78 (1.48, 2.13) <0.001	2.84	4.11 (3.07, 5.51) <0.001	1.30 (0.91, 1.85) 0.153	1.77 (1.25, 2.52) 0.001
As categorical variables (quintiles)								
Q1	7.47	Reference	Reference	Reference	1.37	Reference	Reference	Reference
Q2	8.30	0.97 (0.82, 1.15) 0.760	0.89 (0.77, 1.03) 0.105	0.95 (0.81, 1.11) 0.525	1.76	1.09 (0.76, 1.57) 0.631	0.91 (0.65, 1.28) 0.587	1.05 (0.73, 1.51) 0.796
Q3	11.12	1.23 (1.06, 1.43) 0.006	0.92 (0.80, 1.06) 0.262	0.96 (0.82, 1.12) 0.579	1.97	1.19 (0.93, 1.54) 0.171	0.74 (0.56, 0.99) 0.042	0.87 (0.66, 1.15) 0.326
Q4	14.80	1.57 (1.36, 1.83) <0.001	1.03 (0.89, 1.18) 0.710	1.10 (0.95, 1.28) 0.198	3.30	1.77 (1.29, 2.43) <0.001	0.90 (0.64, 1.26) 0.539	1.19 (0.85, 1.68) 0.312
Q5	27.84	2.64 (2.28, 3.06) <0.001	1.27 (1.08, 1.51) 0.005	1.40 (1.17, 1.67) <0.001	6.11	3.02 (2.30, 3.96) <0.001	1.02 (0.73, 1.42) 0.913	1.34 (0.95, 1.88) 0.094
*P* for trend		<0.001	<0.001	<0.001		<0.001	0.490	0.026

Data are hazard ratios (HRs), 95% confidence intervals (95% CIs), and P-value.

Model I adjust for none.

Model II adjust for age, gender, and race.

Model III adjust for age, gender, race, smoking, SBP, HDL-cholesterol, total cholesterol, eGFR, comorbidities (hypertension, diabetes, and cardiovascular disease), and medicine use (antihypertensive drugs, hypoglycemic agents, lipid-lowering drugs, and antiplatelet drugs).

**Figure 3:**
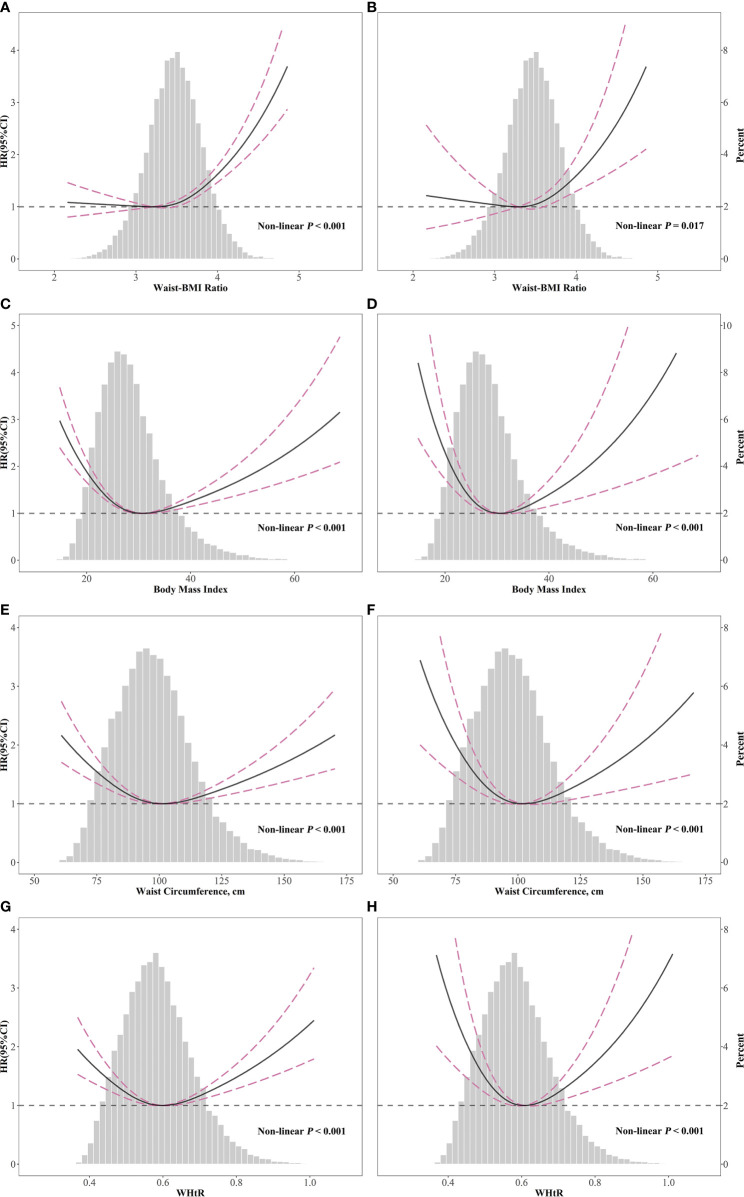
Spline analyses of overall **(A, C, E, G)** and cardiovascular **(B, D, F, H)** mortality by Waist-BMI Ratio **(A, B)**, body mass index **(C, D)**, waist circumference **(E, F)**, and Waist-Height Ratio **(G, H)** and the probability distribution histogram is represented in the background. (Spline analyses were adjusted for age, gender, race, smoking, SBP, HDL-cholesterol, total cholesterol, eGFR, hypertension, diabetes, and cardiovascular disease, antihypertensive drugs, hypoglycemic agents, lipid-lowering drugs, and antiplatelet drugs).

Restricted cubic splines (**Figure 3**) demonstrated a J-shaped relationship of Waist-BMI Ratio with overall (non-linear *P* < 0.001) and cardiovascular mortality (non-linear *P* =0.017), while other anthropometric measures, including BMI, WC, and WtHR, showed asymmetrical U-shaped relationships with mortality (all non-linear *P* < 0.001). Significant differences were detected between the linear regression models and the two-piecewise regression models (log-likelihood ratio test *P*<0.001 for overall mortality and *P*=0.008 for cardiovascular mortality). The cut-off points were estimated by piecewise regression models to be at a Waist-BMI Ratio of 3.72 for overall mortality and 3.66 for cardiovascular mortality (**Table 3**). Above the cut-off points, the risk of overall and cardiovascular death rose steeply with the increase of Waist-BMI Ratio (HR, 3.22; 95% CI, 2.43-4.26; *P* < 0.001 and HR, 3.07; 95% CI, 1.71-5.52; *P* < 0.001, respectively). Nonetheless, no significant association was found below the cut-off points (HR, 1.16; 95% CI, 0.93-1.44; *P* = 0.188 and HR,1.00; 95% CI, 0.58-1.73; *P* = 0.999, respectively).

**Table 3:** The results of two-piecewise linear regression model between Waist-BMI Ratio and cause-specific mortality.

	Overall mortality	Cardiovascular mortality
Cutoff value	3.72	3.66
<Cut-off value	1.16 (0.93, 1.44) 0.188	1.00 (0.58, 1.73) 0.999
≥Cut-off value	3.22 (2.43, 4.26) <0.001	3.07 (1.71, 5.52) <0.001
*P* for log likelihood ratio test	<0.001	0.008

Data are hazard ratios (HRs), 95% confidence intervals (95% CIs), and P-value.

The two-piecewise linear regression model were adjusted for age, gender, race, smoking, SBP, HDL-cholesterol, total cholesterol, eGFR, comorbidities (hypertension, diabetes, and cardiovascular disease), and medicine use (antihypertensive drugs, hypoglycemic agents, lipid-lowering drugs, and antiplatelet drugs).

### The Predictive Value of Waist-BMI Ratio and Other Anthropometric Measures in Overall and Cardiovascular Mortality

**Figure 4:**
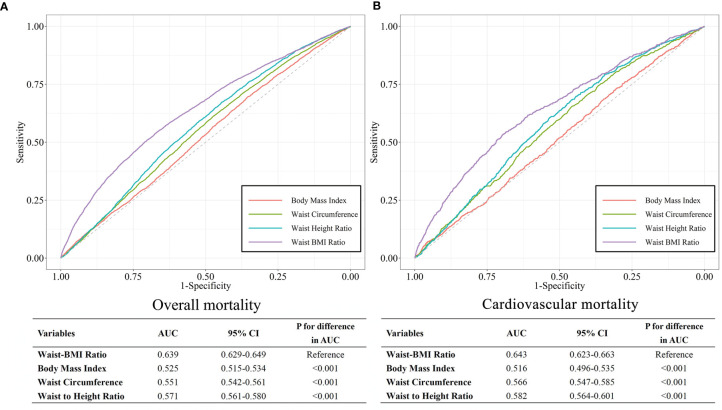
ROC curves for Body mass index, Waist circumference, Waist to Height Ratio, and Waist-BMI Ratio for predicting overall **(A)** and cardiovascular **(B)** mortality.

The ROC curve analysis comparing the predictive ability of different anthropometric measures demonstrated that Waist-BMI Ratio was the strongest predictor of overall mortality (AUC, 0.637; 95% CI, 0.627-0.647). As shown in **Figure 4**, the AUC of BMI, WC, and WtHR for predicting overall mortality were 0.523 (95% CI, 0.513-0.533), 0.552 (95% CI, 0.543-0.562), and 0.572 (95% CI, 0.562-0.581), respectively. Similar results were observed for cardiovascular death. When compared with other indexes, Waist-BMI Ratio showed significantly better performance (AUC, 0.643; 95% CI, 0.623-0.663) than BMI (AUC, 0.516; 95% CI, 0.496-0.535), WC (AUC, 0.566; 95% CI, 0.547-0.585), and WtHR (AUC, 0.582; 95% CI, 0.564-0.601) in predicting cardiovascular death (all *P* for difference in AUC < 0.001). The optimal value of the Waist-BMI Ratio was 3.60 for predicting overall mortality, with a sensitivity of 53.8% and a specificity of 67.4%; as the optimal value was 3.64 for predicting cardiovascular mortality, with a sensitivity of 53.4% and specificity of 69.6%.

## Discussion

In this study, we retrospectively investigated the relationship between a newly defined anthropometric measure and mortality. The results demonstrated that the Waist-BMI Ratio was positively associated with overall and cardiovascular mortality in a J-shaped pattern. Compared with traditional obesity indices, the Waist-BMI Ratio can more adequately predict the risk of death and could be a valuable indicator of the higher-risk phenotype of obesity.

For decades, BMI has been used as an indicator of obesity and has been introduced into various predictive models as a cardiovascular risk factor (25–27). However, a proportion of people with normal BMI have a series of metabolic risk factors, and may be described as “metabolically with obesity but of normal weight” (28), while some individuals with obesity seem to be protected from or more resistant to the development of metabolic abnormalities, and are known as “metabolically healthy but with obesity” (29). One of the reasons may lie in the inability to describe visceral fat and ectopic fat deposition (8), because information from a single anthropometric measure cannot provide sufficient insights into body fat distribution (12). Adverse metabolic effects of excess body fat, including insulin resistance and dyslipidemia, are mainly linked to dysfunctional abdominal subcutaneous adipose tissue and visceral adipose tissue accumulation (9). Experimental models have shown that visceral adipose tissue produces potentially proinflammatory adipokines and macrophage signals, which may be involved in myocardial hypertrophy, fibrosis, and injury (30).

A meta-analysis has revealed that BMI fails to identify half of individuals with excess body fat (31). Chanchal et al. found that the joint use of BMI and WtHR could be conducive to recognizing patients with the highest risk of the composite outcomes (16). A Consensus Statement from the IAS and ICCR Working Group claimed that, although waist circumference is closely linked to overall and cardiovascular death, the full strength of these associations is revealed only after adjustment for BMI (12). However, as a statistical term, “adjustment” might not be easy to understand and use in clinical applications, which limits the combination of WC and BMI. Clinicians have been recommended to evaluate WC variation among patients with similar BMI values, whereas the current obesity-risk classification system still uses the same WC threshold values for all BMI categories (32, 33).

Consistent with previous studies, our present analysis illustrated that individuals in the upper Waist-BMI Ratio quantile, which corresponds to individuals with high WC but low BMI, had the highest risk of mortality. Moreover, patients with low absolute BMI and WC are prone to a higher risk of mortality (34, 35). Studies have indicated that underweight is correlated with undernutrition, inflammation, and other underlying wasting diseases that potentially explain the enhanced risk of death (11, 36, 37). Therefore, U-shape relationships and an “obesity paradox” have often been reported (6, 38). This flaw is circumvented by using Waist-BMI Ratio as an adiposity indicator and thus J-shape relationships were observed. Meanwhile, people of normal weight generally pay less attention to their health indices and do not take preventive measures against obesity-related diseases. Compared with costly and time-consuming imaging-based methods, anthropometric methods such as the Waist-BMI Ratio form a more convenient, comprehensible, and even home self-testing monitoring system for early identification of high-risk individuals and for disease prevention.

Our study had several notable advantages. First, the study was based on the NHANES dataset, a nationally representative survey with rigorous methodology and comprehensive quality control procedures; the large sample size was sufficient to provide good statistical power. Second, our analysis was adjusted for study weights and the complex survey design to reduce estimation errors. Third, the newly defined parameter was easily obtained and calculated, with a clear and unilateral risk threshold. Nevertheless, several limitations pertain to our study. First, we were unable to obtain accurate information about body composition. Therefore, we could not calculate the correlation coefficient between Waist-BMI Ratio and abdominal or visceral adipose tissue. Second, definitive causal inferences cannot be drawn because of the observational nature of this study. Third, although our analyses controlled for important confounding variables, the possibility of residual confounding variables remains. For example, we were unable to adjust for alcohol consumption and physical activity level due to missing covariate data. Fourth, death certificates may not precisely represent the real cause of death. Fifth, NHANES data are representative of the United States population, thus probably limiting the applicability of our results to other regions and ethnic populations.

## Conclusion

As a newly defined anthropometric measure, Waist-BMI Ratio was independently associated with overall and cardiovascular mortality after mutual adjustment. Compared with other traditional anthropometric measures, Waist-BMI Ratio had a better predictive ability and a more certain risk threshold value for mortality. Because it is convenient, easy to access, and virtually free cost, Waist-BMI Ratio can be used as a valuable indicator for the high-risk phenotype of obesity.

## Data Availability Statement 

Publicly available datasets were analyzed in this study. This data can be found here: https://www.cdc.gov/nchs/nhanes/index.htm.

## Ethics Statement

The studies involving human participants were reviewed and approved by the Institutional Review Board of the Centers for Disease Control and Prevention (Protocol 98–12, 2005–06 and 2011–17). The patients/participants provided their written informed consent to participate in this study.

## Author Contributions

Conceptualization, XCL and YQH. Methodology, XCL. Validation, YQF, YQH and JYC. Formal Analysis, XCL. Investigation, KL. Resources, YQF. Data Curation, XCL. Writing — Original Draft Preparation, XCL. Writing – Review and Editing, YH. Visualization, XCL. Supervision, JYC. Project Administration, YQF. Funding Acquisition, YQF. All authors contributed to the article and approved the submitted version.

## Funding

This research was funded by the National Key Research and Development Program of China (No. 2017YFC1307603), the Science and Technology Plan Program of Guangzhou (No. 201803040012), the Key Area R&D Program of Guangdong Province (No. 2019B020227005), Guangdong Provincial People’s Hospital Clinical Research Fund (Y012018085), the Fundamental and Applied Basic Research Foundation Project of Guangdong Province (2020A1515010738), and the Climbing Plan of Guangdong Provincial People’s Hospital (DFJH2020022).

## Conflict of Interest

The authors declare that the research was conducted in the absence of any commercial or financial relationships that could be construed as a potential conflict of interest.
